# Hypnotic and sleep-promoting effects of *Limosilactobacillus reuteri* LM1063 on pentobarbital-induced sleep and electroencephalogram analysis in mice

**DOI:** 10.1038/s41598-026-42833-0

**Published:** 2026-03-09

**Authors:** Min Gyeong Kim, Eunsol Seo, Ju Young Eor, Anna Kang, Tae Rahk Kim, Minn Sohn, Younghoon Kim

**Affiliations:** 1 Department of Agricultural Biotechnology and Research Institute of Agriculture and Life Science, Seoul National University, Seoul, 08826 Korea; 2 LactoMason Co., Ltd., Jinju, 52840 Korea

**Keywords:** Probiotics, Gut-brain axis, Sleep architecture, Sleep health, *Limosilactobacillus reuteri* LM1063, Neuroscience, Physiology

## Abstract

Sleep disturbances are an increasing health concern, and limitations associated with long-term use of conventional hypnotics have prompted interest in alternative approaches that support sleep health. Growing evidence suggests that the gut–brain axis contributes to sleep regulation; however, the effects of probiotics on objective sleep parameters and their underlying mechanisms remain incompletely understood and may vary across probiotic strains. The present study investigated the sleep**–**modulating effects of a selected probiotic strain, *Limosilactobacillus reuteri* LM1063 (LM1063), using murine models. Sleep latency and duration were assessed using a pentobarbital-induced sleep test, and sleep architecture was evaluated by electroencephalogram (EEG) recordings, including rapid eye movement (REM) and non-rapid eye movement (NREM) sleep. To explore potential mechanisms, neurochemical analyses focusing on key neurotransmitters, including gamma-aminobutyric acid (GABA) and serotonin, were conducted. Administration of LM1063 significantly shortened sleep latency and prolonged sleep duration in mice. EEG analysis revealed shifts in overall sleep architecture toward a sleep-favorable state without disruption of normal sleep organization. These sleep-related changes were accompanied by modulation of GABAergic and serotonergic neurochemical pathways. In conclusion, LM1063 exerts strain-specific sleep-modulating effects through neurochemical mechanisms associated with the gut–brain axis. By integrating behavioral sleep assessment, electrophysiological analysis, and neurochemical profiling, this study provides mechanistic insight supporting the potential translational application of microbiome-targeted approaches for promoting sleep health.

**Supplementary Information:**

The online version contains supplementary material available at 10.1038/s41598-026-42833-0.

## Introduction

 Sleep disorders, particularly insomnia, are a growing public health concern worldwide due to their substantial impact on physical health, cognitive performance, and overall quality of life^1^. Although conventional pharmacological treatments, such as benzodiazepines and melatonin supplements, are widely used and generally effective, their long-term use is often limited by adverse effects, tolerance, and dependence^1,2^. These limitations have prompted increasing interest in alternative or adjunctive approaches that may improve sleep with a more favorable safety profile.

In this context, the gut–brain axis has emerged as an important regulatory system linking the central nervous system with the intestinal microbiota. Accumulating evidence suggests that gut microorganisms influence sleep–wake regulation through multiple pathways, including the modulation of neurotransmitter synthesis, immune signaling, and metabolic homeostasis^3,4^. In particular, gut-derived metabolites and neuroactive compounds such as gamma-aminobutyric acid (GABA) and serotonin have been implicated in the regulation of sleep architecture and circadian rhythms^4,5^. Despite growing interest in this field, microbiota-targeted strategies for sleep improvement remain less well characterized than conventional hypnotic therapies, especially with respect to objective sleep parameters and underlying mechanisms.

Probiotics, live microorganisms conferring health benefits when administered adequately, have shown promise in positively shaping the gut microbiota and exerting neuropsychological effects relevant to sleep and mood disorders^6,7^. Several randomized controlled trials have reported improvements in subjective sleep quality, most commonly assessed by the Pittsburgh Sleep Quality Index (PSQI), as well as reductions in anxiety and depressive symptoms following probiotic supplementation^6–8^. Consistent with these findings, recent meta-analyses indicate that probiotics can produce modest but clinically meaningful improvements in PSQI scores among individuals with insomnia or poor sleep quality^9,10^. However, effects on objective sleep measures, such as total sleep time and sleep efficiency, remain inconsistent across studies, highlighting the need for controlled experimental models to clarify the neurobiological mechanisms underlying these observations.

Importantly, accumulating evidence indicates that probiotic effects on sleep are strain-specific. Clinical studies have demonstrated that *Bifidobacterium animalis* subsp. *lactis* BLa80 improves subjective sleep quality and increases GABA levels in a double-blind randomized trial, whereas *Lactobacillus plantarum* PS128 has been shown to modulate electroencephalogram (EEG)-based sleep parameters and alleviate depressive symptoms in individuals with insomnia^7,11^. Together, these findings emphasize the importance of evaluating probiotic effects at the strain level using objective and mechanistically informative approaches. Nevertheless, objective metrics such as total sleep time and sleep efficiency show heterogeneous results across trials, reinforcing the need for preclinical models capable of probing neurotransmitter pathways, including GABAergic and serotonergic modulation^6^.

Building on this foundation, the present study focuses on investigating the effects of a selected strain, *Limosilactobacillus reuteri* LM1063 (LM1063), on sleep acceleration and sleep architecture in murine models. Pentobarbital-induced sleep tests were employed to quantify sleep latency and duration, while EEG recordings provided detailed assessments of sleep stages including rapid eye movement (REM) and non-rapid eye movement (NREM). In line with the sleep-related mechanisms explored in the present study, previous animal studies have reported that administration of *Bifidobacterium breve* induces shifts in overall sleep architecture toward a sleep-favorable state via gut–brain signaling, accompanied by modulation of central GABAergic and serotonergic pathways, thereby supporting strain-specific modulation of sleep architecture beyond subjective measures^12^. In a similar mechanistic context, studies employing pharmacological sleep models have demonstrated that sleep initiation and maintenance can be improved through modulation of GABAergic signaling. Specifically, Lemon Verbena Extract was shown to reduce sleep latency and increase total sleep time in pentobarbital-induced and polysomnography-based models via regulation of adenosine A1 and GABAA receptors^13^. These findings provide additional evidence that sleep improvement can be achieved through GABAergic mechanisms, paralleling those implicated in probiotic-mediated effects.

Accordingly, the present study incorporated neurochemical analyses focusing on key neurotransmitters, including GABA and serotonin, to explore potential mechanisms underlying the sleep-modulating effects of strain-specific probiotics. By integrating behavioral sleep assessments with neurochemical measurements, this study provides mechanistic insight that supports the potential translational application of microbiome-targeted approaches for promoting sleep health.

## Results

### Effect of probiotic administration on sleep latency and duration in a pentobarbital-induced sleep model

**Fig. 1:**
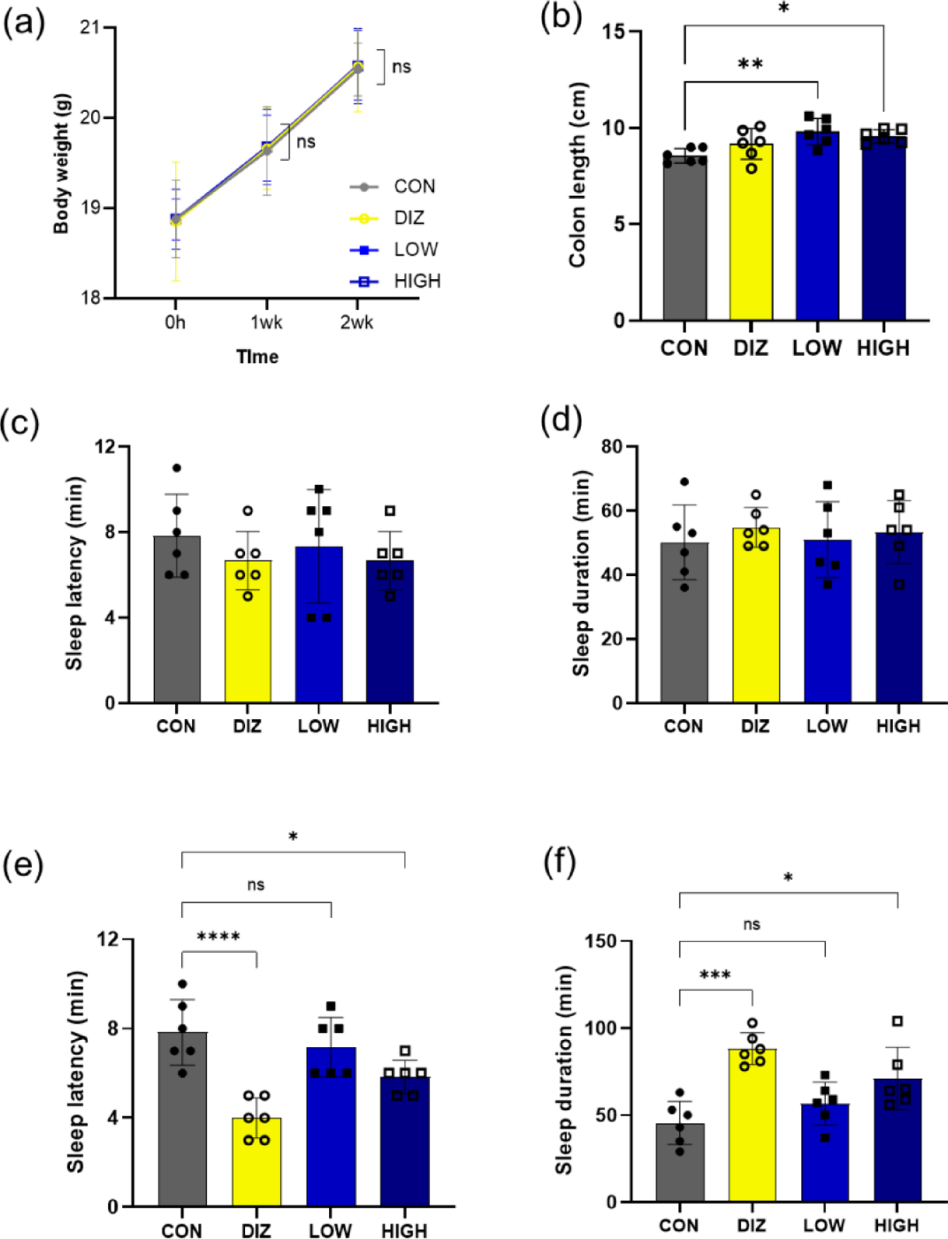
Effects of probiotic supplementation on physiological and sleep parameters in the pentobarbital-induced sleep model. **(a)** Body weight measured throughout the experimental period. **(b)** Intestinal length quantified immediately after dissection using ImageJ software. **(c-d)** Baseline sleep latency and sleep duration. **(e-f)** Sleep latency and sleep duration after 14days of probiotic administration. Data are mean ± SEM (*n* = 6 per group). *p < 0.05, **p < 0.01, ***p < 0.001, ****p < 0.0001 vs. CON (one-way ANOVA, Tukey’s post hoc test). CON: control (PBS), DIZ: positive control (diazepam 2 mg/kg), LOW: low-dose LM1063 (1 × 10⁹ CFU), and HIGH: high-dose LM1063 (1 × 10¹⁰ CFU).

During the 2-week experimental period, body weight remained stable across all groups, with no significant differences compared with the CON group (Fig. 1a). Daily food intake was also consistent among groups (approximately 3.7–3.9 g/mouse/day), indicating that probiotic administration did not influence general metabolism or feeding behavior. In contrast, intestinal length measured after dissection was significantly increased in probiotic-treated mice (Fig. 1b). Both the LOW and HIGH groups exhibited longer colons compared with the CON group (*p* < 0.01 and *p* < 0.05, respectively), while the DIZ group showed a similar upward trend without statistical significance. These results suggest that probiotic supplementation may contribute to intestinal health and tissue maintenance without affecting overall growth or food consumption.

Before probiotic administration, no significant differences in sleep latency or sleep duration were observed among the groups (Fig. 1c–d), confirming comparable baseline sleep responsiveness. Although a slight increase in sleep duration was observed in the DIZ group even at baseline, which may reflect the intrinsic sedative property of diazepam; however, this difference was minimal and did not reach statistical significance. After 14 days of administration, the analysis of sleep latency revealed a significant reduction in both the DIZ group and HIGH groups compared with the CON group. Specifically, sleep latency was markedly shortened in the DIZ group (*p* < 0.0001) and moderately reduced in the HIGH group (*p* < 0.05) (Fig. 1e). Similarly, sleep duration was significantly prolonged in the DIZ (*p* < 0.001) and HIGH (*p* < 0.05) groups, with a mild but nonsignificant increase observed in the LOW group (Fig. 1f).

### Changes in sleep pattern and sleep architecture following probiotic administration

**Fig. 2:**
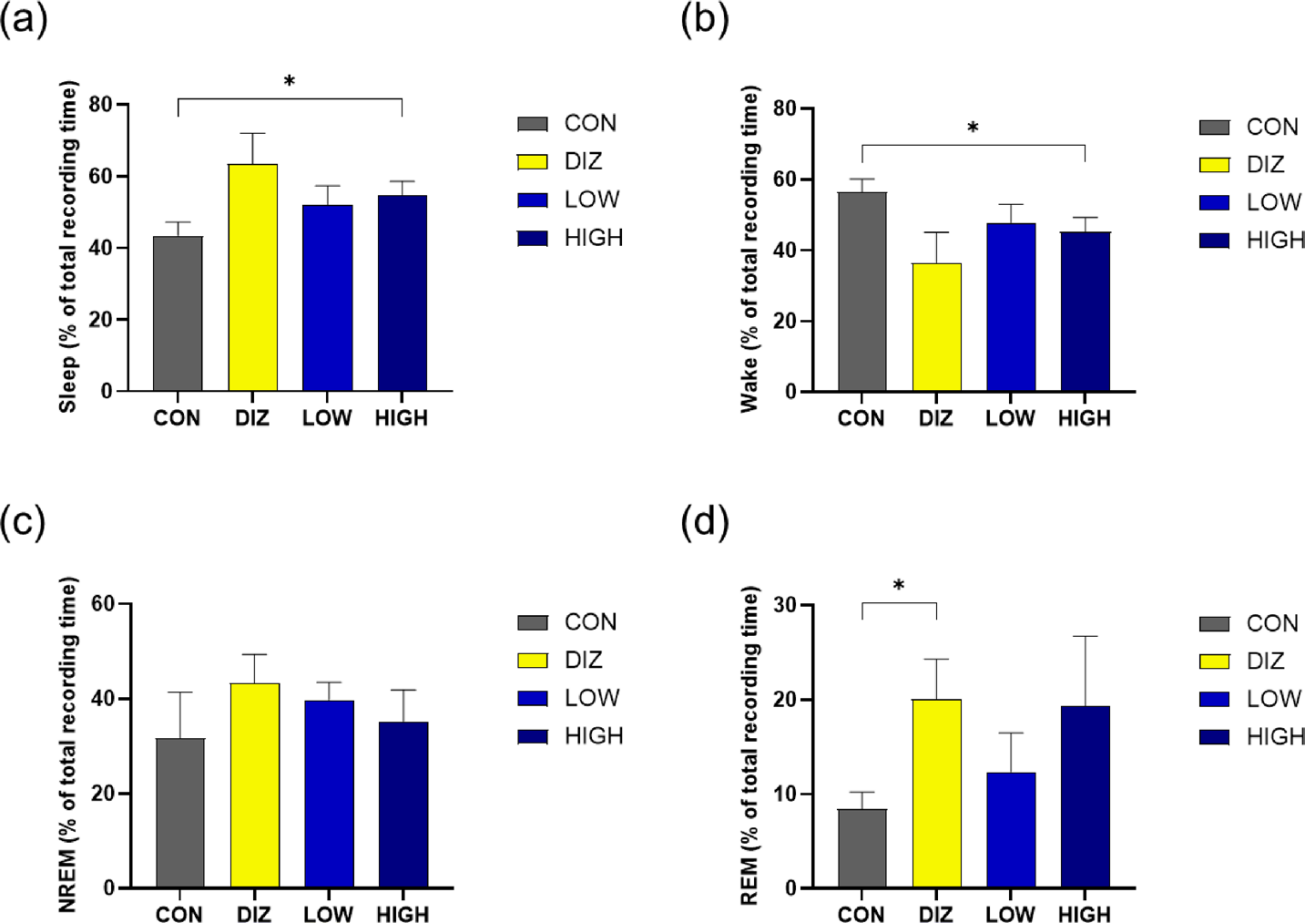
EEG/EMG-based analysis of sleep-wake ratio and sleep architecture after probiotic administration. **(a)** Sleep ratio **(b)** wakefulness ratio **(c)** NREM sleep proportion and **(d)** rapid eye REM sleep proportion analyzed from the total EEG recording time. Data are presented as mean ± SEM (*n* = 5 per group). *p < 0.05 vs. CON (one-way ANOVA with Tukey’s post hoc test). CON: control (PBS), DIZ: positive control (diazepam 2 mg/kg), LOW: low-dose LM1063 (1 × 10⁹ CFU), and HIGH: high-dose LM1063 (1 × 10¹⁰ CFU).

EEG/EMG analysis was conducted at both baseline and after 14 days of administration to evaluate the proportion of sleep and wakefulness relative to the total recording time. At baseline, no significant differences in sleep or wakefulness ratios were observed among the groups, indicating comparable baseline sleep architecture (Table 1). Two-way ANOVA with Group and Time as factors revealed a significant main effect of Time on both sleep and wakefulness ratios (*p* = 0.0006), whereas neither the main effect of Group nor the Group × Time interaction reached statistical significance, indicating an overall shift toward increased sleep and decreased wakefulness over time across groups. After 14 days of administration, compared with the CON group, the HIGH group showed a significant increase in the proportion of sleep and a significant decrease in the proportion of wakefulness (*p* < 0.05). Although not statistically significant, both the DIZ and LOW groups exhibited a trend toward increased sleep and decreased wakefulness (Fig. 2a–b; Table 1).

**Table 1:** Sleep and wakefulness ratios at baseline and after 14 days of probiotic administration.

	Baseline (0 d)		14 d	
	Sleep (%)	Wake (%)	Sleep (%)	Wake (%)
CON	42.30 ± 11.11	57.70 ± 11.11	43.51 ± 3.70	56.49 ± 3.70
DIZ	39.44 ± 15.96	60.56 ± 15.96	63.46 ± 8.56	36.54 ± 8.56
LOW	49.98 ± 7.85	69.02 ± 7.85	52.16 ± 5.23	47.84 ± 5.23
HIGH	40.97± 18.16	59.03 ± 18.16	54.69 ± 3.97	45.31 ± 3.97

CON: control (PBS), DIZ: positive control (diazepam 2 mg/kg), LOW: low-dose LM1063 (1 × 10⁹ CFU), and HIGH: high-dose LM1063 (1 × 10¹⁰ CFU). Two-way ANOVA revealed a significant main effect of Time for both sleep and wakefulness ratios (*p* = 0.0006), whereas no significant Group × Time interaction was detected.

**Table 2:** Proportions of NREM and rapid eye movement REM sleep at baseline and after 14 days of probiotic administration.

	Baseline (0 d)		14 d	
	NREM (%)	REM (%)	NREM (%)	REM (%)
CON	34.99 ± 12.36	7.04 ± 1.66	31.75 ± 9.63	8.43 ± 1.81
DIZ	28.75 ± 12.32	10.64 ± 4.20	43.29 ± 6.08	20.03 ± 4.28
LOW	30.81 ± 5.93	9.14 ± 2.73	39.76 ± 3.71	12.37 ± 4.12
HIGH	33.46 ± 16.29	7.44 ± 1.97	35.22 ± 6.64	19.37 ± 7.34

CON: control (PBS), DIZ: positive control (diazepam 2 mg/kg), LOW: low-dose LM1063 (1 × 10⁹ CFU), and HIGH: high-dose LM1063 (1 × 10¹⁰ CFU). Two-way ANOVA with Group and Time as factors revealed a significant Group × Time interaction for REM sleep proportion (*p* = 0.0158), along with significant main effects of Time (*p* < 0.0001) and Group (*p* = 0.0009). In contrast, no significant main effects or interactions were detected for NREM sleep proportion.

Sleep architecture analysis was subsequently conducted by calculating the proportions of REM and NREM sleep relative to the total EEG/EMG recording time. At baseline, the relative proportions of REM and NREM sleep did not differ significantly among groups, with NREM sleep accounting for a higher proportion of total sleep than REM sleep across all groups (Table 2). After 14 days of administration, the overall predominance of NREM sleep was maintained across groups. Two-way ANOVA with Group and Time as factors revealed a significant Group × Time interaction for REM sleep proportion (*p* = 0.0158), along with significant main effects of Time (*p* < 0.0001) and Group (*p* = 0.0009), indicating differential modulation of REM sleep across treatment groups over time. In contrast, no significant main effects or interaction were detected for NREM sleep proportion. No significant differences in NREM sleep ratios were observed among the groups (Fig. 2c), whereas the DIZ group exhibited a significantly higher proportion of REM sleep compared with the CON group (*p* < 0.05). Both the HIGH and LOW groups showed a nonsignificant tendency toward increased REM sleep (Fig. 2d).

### Expression of GABA-related genes following probiotic administration

**Fig. 3:**
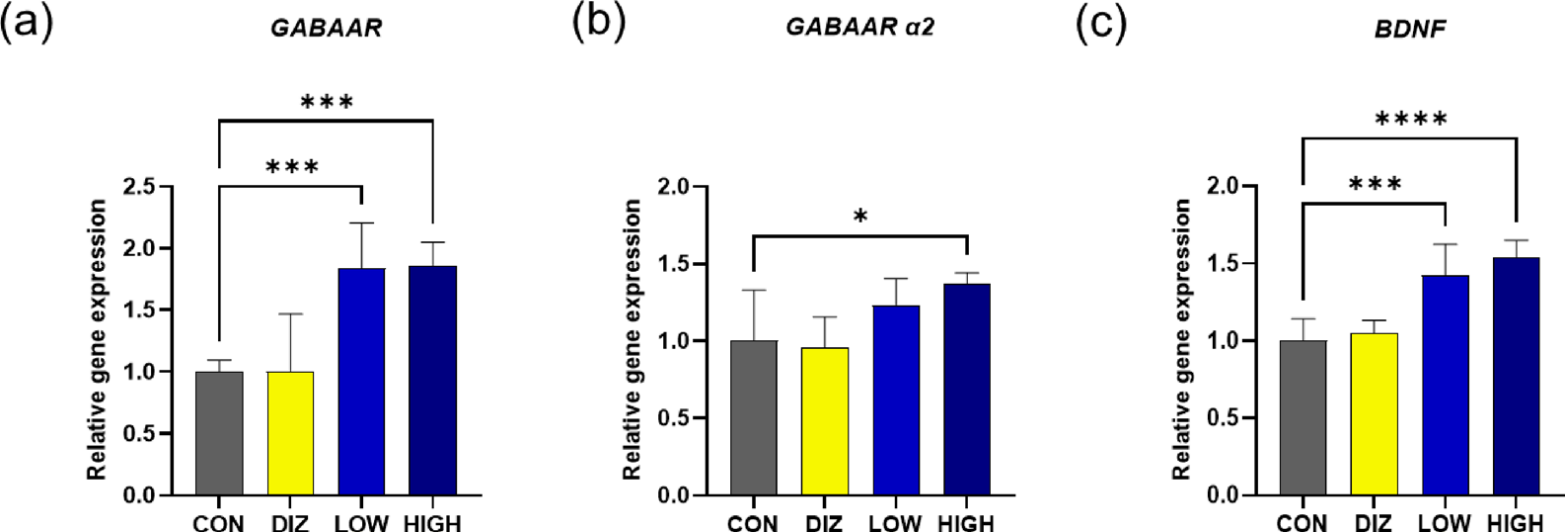
Relative expression of neurotransmission-related genes in brain tissue after probiotic administration. **(a)** GABA receptor genes **(b)** GABA receptor α2 subunit **(c)** BDNF. Data are presented as mean ± SEM (*n* = 6 per group). *p < 0.05, **p < 0.01, ***p < 0.001 vs. CON (one-way ANOVA with Tukey’s post hoc test). CON: control (PBS), DIZ: positive control (diazepam 2 mg/kg), LOW: low-dose LM1063 (1 × 10⁹ CFU), and HIGH: high-dose LM1063 (1 × 10¹⁰ CFU).

Total RNA extracted from the midbrain and hippocampus, was analyzed using RT-qPCR. The overall expression of GABA receptor gene (*GABAAR*) was significantly increased in both the LOW and HIGH groups compared with the CON group (*p* < 0.001) (Fig. 3a). In particular, the expression of the GABA receptor α2 subunit gene (*GABAA​R α2*) was significantly upregulated in the HIGH group compared with the CON group (*p* < 0.05) (Fig. 3b). In addition, the expression of brain-derived neurotrophic factor (BDNF) was significantly elevated in both the LOW and HIGH groups relative to the CON group (*p* < 0.001) (Fig. 3c), suggesting enhanced synaptic plasticity associated with increased GABAergic activity.

### Expression of serotonin-related genes following probiotic administration

**Fig. 4:**
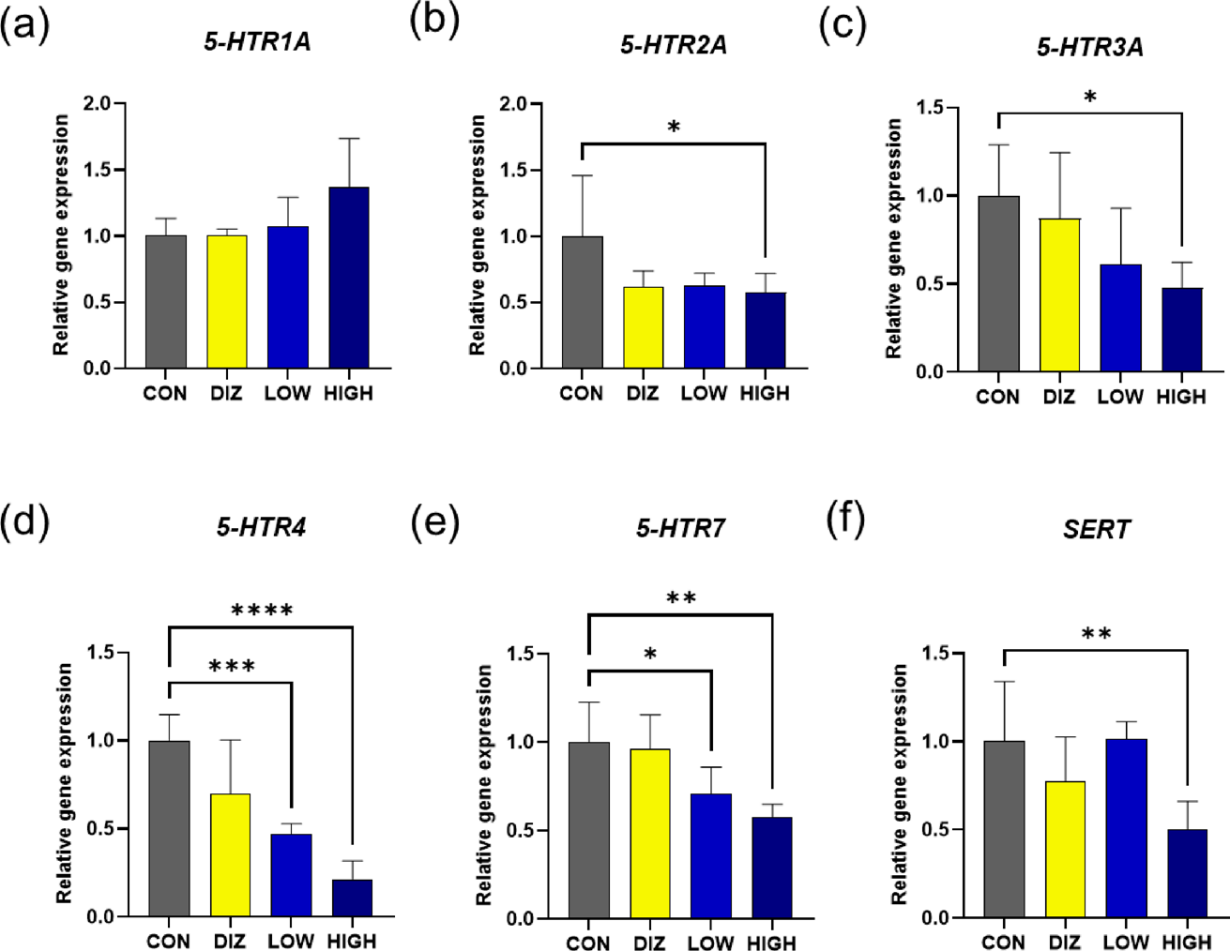
Relative expression of serotonin (5-hydroxytryptamine, 5-HT) receptor–related genes in brain tissue after probiotic administration. **(a)** 5-HTR1A **(b)** 5-HTR2A **(c)** 5-HTR3A **(d)** 5-HTR4 **(e)** 5-HTR7 **(f)** SERT. Data are presented as mean ± SEM (*n* = 6 per group). *p < 0.05, **p < 0.01, ***p < 0.001 vs. CON (one-way ANOVA with Tukey’s post hoc test). CON: control (PBS), DIZ: positive control (diazepam 2 mg/kg), LOW: low-dose LM1063 (1 × 10⁹ CFU), and HIGH: high-dose LM1063 (1 × 10¹⁰ CFU).

Similarly, analysis of serotonin-related gene expression revealed that the HIGH group exhibited significantly reduced expression of serotonin receptor genes involved in arousal and sensory processing, including *5-HTR2A*, *5-HTR3A*, *5-HTR4*, and *5-HTR7* (*p* < 0.05–0.001) (Fig. 4b-e). In contrast, the expression of the *5-HTR1A* gene, which is implicated in emotional stabilization and anxiolytic effects, showed a nonsignificant but increasing trend (Fig. 4a). The serotonin transporter gene (*SERT*) was also significantly downregulated in the HIGH group (*p* < 0.01) (Fig. 4f), suggesting that probiotic administration may attenuate serotonergic signaling related to arousal while maintaining the stabilizing components of the serotonin pathway.

### Serum glutamate, GABA, and serotonin levels following probiotic administration

**Fig. 5:**
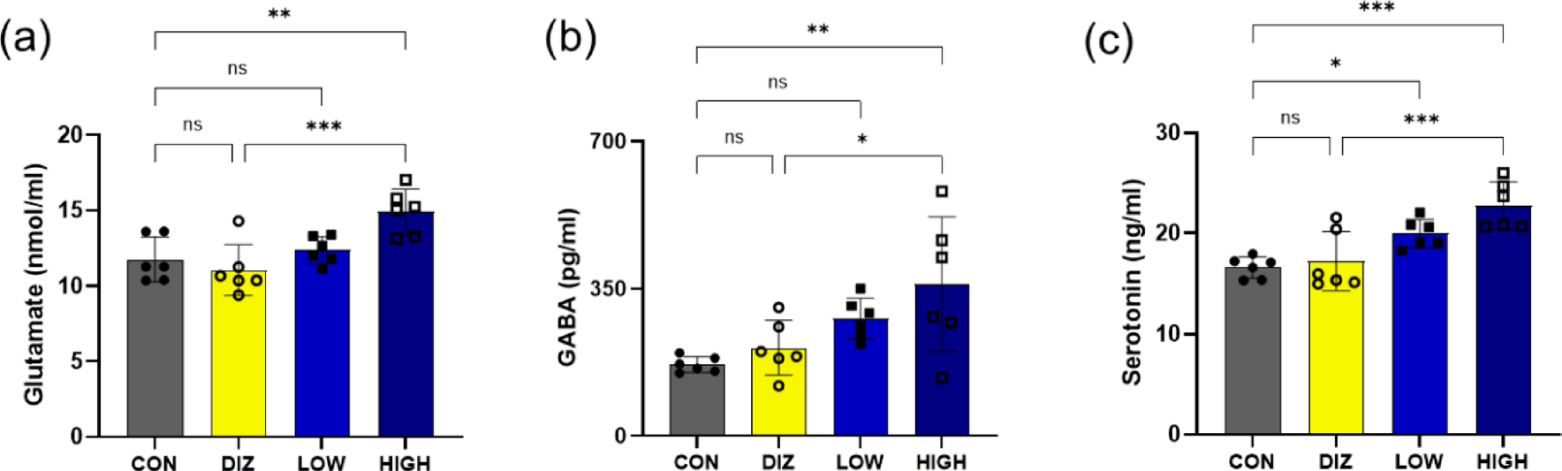
Serum levels of neurotransmitters after probiotic administration. **(a)** Glutamate **(b)** GABA **(c)** Serotonin levels measured by ELISA. Data are presented as mean ± SEM (*n* = 6 per group). *p < 0.05, **p < 0.01, ***p < 0.001 vs. CON (one-way ANOVA with Tukey’s post hoc test). CON: control (PBS), DIZ: positive control (diazepam 2 mg/kg), LOW: low-dose LM1063 (1 × 10⁹ CFU), and HIGH: high-dose LM1063 (1 × 10¹⁰ CFU).

After 14 days of administration, ELISA analysis revealed coordinated changes in circulating neuroactive molecules related to sleep regulation. Serum glutamate levels, representing the metabolic precursor of GABA, were significantly elevated in the HIGH group compared with the CON group (*p* < 0.01) (Fig. 5a). Consistent with this, serum GABA levels were markedly increased in the HIGH group compared with the CON group (*p* < 0.01). Although the DIZ and LOW groups did not differ significantly from the control, both showed a mild upward trend, and the HIGH group also exhibited significantly higher GABA concentrations than the DIZ group (*p* < 0.05) (Fig. 5b).

Similarly, serum serotonin levels tended to increase across all treatment groups after 14 days of administration. A significant elevation was observed in the HIGH group compared with both the CON and DIZ groups (*p* < 0.001) (Fig. 5c).

### Changes in gut microbiome composition following probiotic administration

**Fig. 6:**
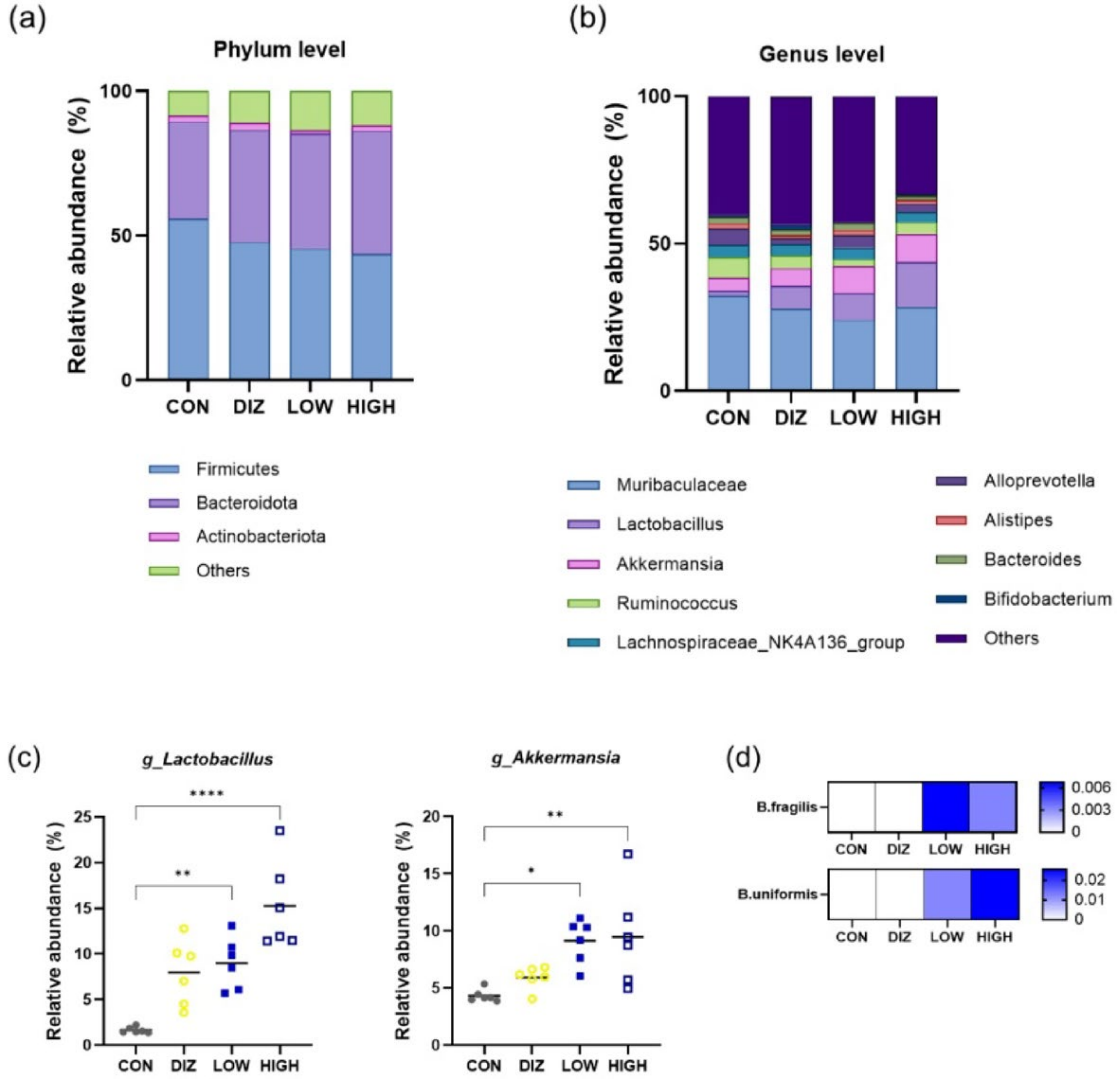
Gut microbiota composition at the phylum and genus levels after probiotic administration. **(a)** Phylum-level and **(b)** genus-level distributions of gut microbiota based on 16S rRNA sequencing. **(c)** Relative abundance of representative taxa including *Lactobacillus* and *Akkermansia*. **(d)** Heatmap showing the relative abundance of *Bacteroides fragilis*, and *Bacteroides uniformis*. Data are presented as mean ± SEM (*n* = 6 per group). CON: control (PBS), DIZ: positive control (diazepam 2 mg/kg), LOW: low-dose LM1063 (1 × 10⁹ CFU), and HIGH: high-dose LM1063 (1 × 10¹⁰ CFU).

Phylum-level taxonomic profiling revealed that the gut microbiota in all groups was predominantly composed of *Firmicutes* and *Bacteroidota*, with smaller proportions of *Actinobacteria* (Fig. 6a). Although the overall phylum-level composition remained largely consistent among groups, the relative abundance of *Bacteroidota* slightly increased in both probiotic-treated groups (LOW and HIGH), accompanied by a modest decrease in *Firmicutes* compared with the CON group.

At the genus level, compositional analysis indicated that *Lactobacillus* and *Akkermansia* were more abundant in the probiotic-treated groups, reflecting the phylum-level shift observed above (Fig. 6b). Quantitative analysis further confirmed that the relative abundances of *Lactobacillus* and *Akkermansia* were significantly elevated in both the LOW and HIGH groups compared with the CON group (Fig. 6c). Additionally, *Bacteroides fragilis* and *Bacteroides uniformis* exhibited an upward trend in both treatment groups, although these changes did not reach statistical significance (Fig. 6d).

### Analysis of gut microbial diversity and community structure (Alpha/Beta diversity)

**Fig. 7:**
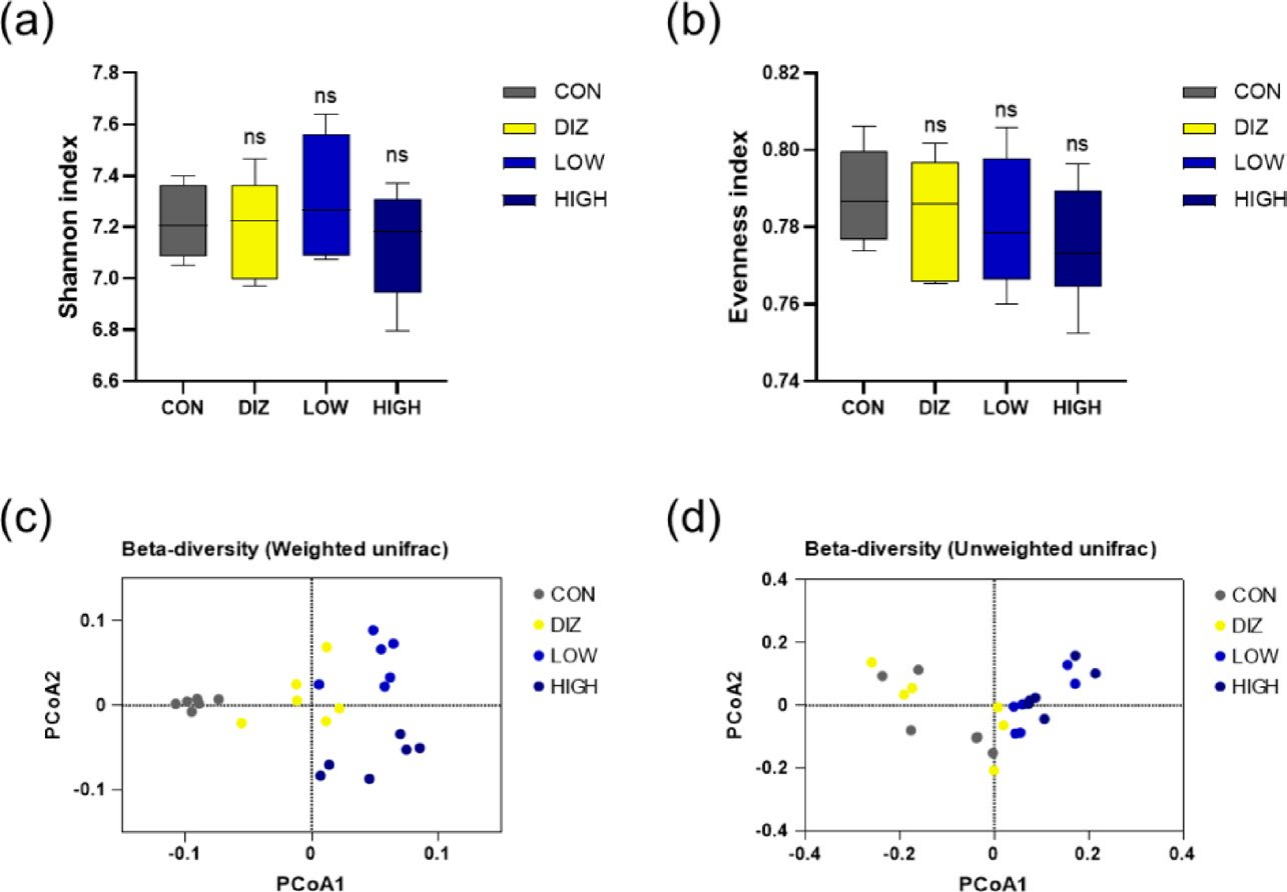
Diversity and differential taxa analysis of gut microbiota following probiotic administration. **(a)** Shannon index and **(b)** evenness index representing α-diversity of the gut microbial community. **(c–d)** PCoA plots based on weighted and unweighted UniFrac distances showing β-diversity among groups. Data are presented as mean ± SEM (*n* = 6 per group). CON: control (PBS), DIZ: positive control (diazepam 2 mg/kg), LOW: low-dose LM1063 (1 × 10⁹ CFU), and HIGH: high-dose LM1063 (1 × 10¹⁰ CFU).

Analysis of α-diversity indices, including Shannon index and evenness, showed no statistically significant differences among the groups (Fig. 7a–b). This suggests that probiotic administration did not cause abrupt alterations in overall microbial diversity or community evenness.

In β-diversity analysis using weighted and unweighted UniFrac-based principal coordinate analysis (PCoA), both probiotic-treated groups (LOW and HIGH) tended to form clusters separated from the CON group (Fig. 7c–d). Notably, the weighted UniFrac results, which account for relative abundance, demonstrated a clearer separation of the treatment groups from the control, suggesting that probiotic supplementation can alter the composition and proportional balance of the gut microbiota.

### Differential gut microbial taxa identified by LEfSe analysis

**Fig. 8:**
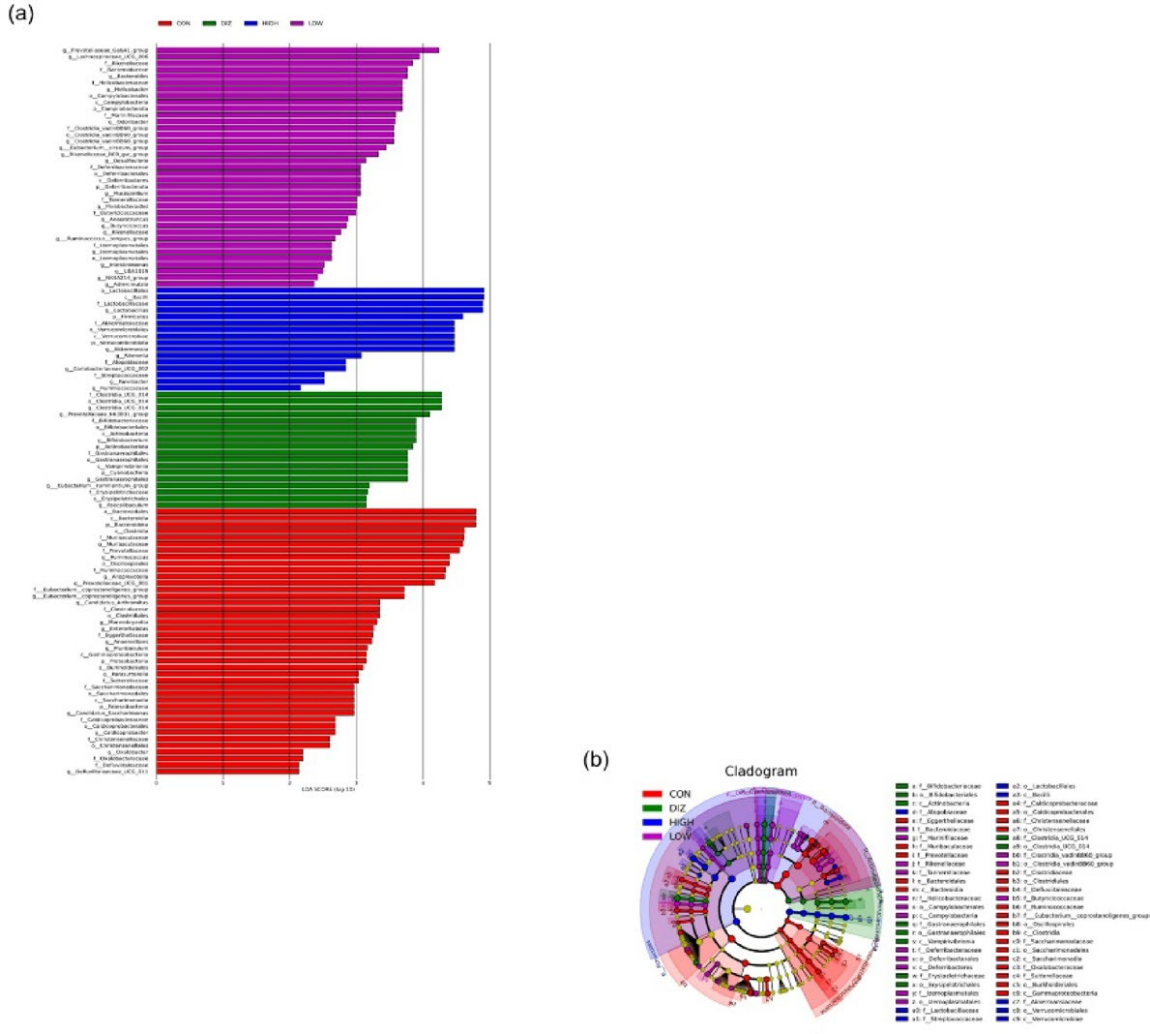
LEfSe identifying differential bacterial taxa among experimental groups. **(a)** Histogram of LDA scores showing bacterial taxa with significantly different relative abundances among groups. **(b)** Cladogram illustrating the phylogenetic distribution of taxa identified by LEfSe analysis. Taxa with LDA scores (log₁₀) > 2.0 were considered significantly enriched. CON: control (PBS), DIZ: positive control (diazepam 2 mg/kg), LOW: low-dose LM1063 (1 × 10⁹ CFU), and HIGH: high-dose LM1063 (1 × 10¹⁰ CFU).

LEfSe analysis revealed that the relative abundances of *Akkermansiaceae* (*Akkermansia*) and *Lactobacillaceae* (*Lactobacillus*) were significantly increased in the HIGH group compared with the CON group (Fig. 8a–b). These findings are consistent with the trends observed in the heatmap and compositional analyses, providing additional statistical evidence that probiotic administration promotes the proliferation of beneficial gut bacteria.

As a complementary analysis, RT-qPCR analysis of intestinal tissues (colonic region) was performed for intestinal barrier-related genes (*Occludin*, *Claudin-1*, and *ZO-1*) and the mucus-related gene *Muc2*, and the results are provided in Supplementary Fig. S1.

## Discussion

In this study, we demonstrated that probiotic supplementation improved sleep parameters in mice, accompanied by coordinated changes in neurochemical and microbial pathways. The pentobarbital-induced sleep model was employed as a standardized pharmacological assay to sensitively assess sleep-potentiating activity, whereas physiological sleep architecture was evaluated using EEG/EMG recordings. The pentobarbital-induced sleep assay revealed that probiotics significantly shortened sleep latency and prolonged sleep duration, indicating an enhancement of sleep initiation and maintenance capacity. These behavioral improvements were corroborated by EEG and EMG analyses, which showed that probiotic administration, particularly at high doses, increased total sleep ratio and reduced wakefulness compared with the control group. Moreover, the proportion of REM sleep was higher in probiotic-treated mice, while NREM sleep remained relatively stable. Given that GABAergic circuits contribute to the regulation of REM sleep transitions through inhibitory control within the pontine and medullary regions^14^, and that serotonergic signaling plays a modulatory role in arousal regulation^15^, the REM-associated changes observed here likely reflect a broader neurochemical environment permissive to sleep continuity rather than a direct, REM-specific effect. Consistent with this interpretation, molecular analyses revealed coordinated modulation of GABAergic and serotonergic markers, supporting an indirect contribution to sleep structure stabilization.

At the molecular level, probiotic supplementation upregulated the expression of the GABA receptor and its α2 subunit, suggesting potential strengthened GABAergic signaling that may reduce neural excitability and facilitate sleep. The specific increase in the α2 subunit is particularly noteworthy, as this isoform predominates in limbic regions and mediates anxiolytic and relaxing effects without inducing strong sedation^16^. This suggests that probiotics may promote sleep in part by alleviating anxiety and enhancing emotional stability. Concurrent elevation of *BDNF* expression further supports this mechanism, as *BDNF*-driven plasticity within GABAergic circuits contributes to stress resilience and long-term maintenance of sleep quality^17,18^.

Probiotics also modulated serotonergic signaling in a direction consistent with reduced arousal and improved emotional regulation. Several arousal-promoting serotonin receptors (*5-HTR2A*, *5-HTR3A*, *5-HTR4*, *5-HTR7*) were downregulated in the HIGH group, while *5-HTR1A*, associated with anxiolytic effects, showed a mild increase^15,19^. The suppression of *5-HTR2A* is especially relevant, as this receptor facilitates cortical activation and wakefulness^20,21^ its downregulation may thus favor REM sleep expression^22^.

In parallel, the observed reduction in *SERT* expression may have limited serotonin reuptake, allowing serotonin to remain longer in the synaptic space. While synaptic dynamics were not directly measured, sustained serotonin availability could contribute to the elevated systemic serotonin levels detected in the probiotic-treated groups. Previous studies have shown that downregulation of the serotonin transporter markedly reduces serotonin clearance and prolongs its synaptic presence^23^, while chronic increases in extracellular serotonin can induce compensatory downregulation or desensitization of excitatory serotonin receptors^24,25^. Such adaptive regulation helps prevent overstimulation and may dampen arousal-related serotonergic signaling, thereby promoting a calmer and more sleep-permissive neurochemical environment. Together, these transcriptional adjustments suggest that probiotics rebalance serotonergic networks toward a calmer, sleep-permissive neurochemical state.

Biochemical measures further supported these findings. ELISA analysis showed increased serum concentrations of glutamate, GABA, and serotonin after probiotic supplementation. Given that glutamate serves as a metabolic precursor for GABA synthesis, concurrent elevations of both metabolites suggests modulation of peripheral glutamate–GABA metabolism rather than direct enhancement of central inhibitory neurotransmission. Similarly, increased serum serotonin, which is predominantly produced in the gastrointestinal tract, is more likely to reflect altered peripheral neurochemical states associated with gut–brain axis signaling than direct serotonergic neurotransmission in the brain. Together, these data indicate that probiotic administration induces systemic neurochemical changes that may contribute to a sleep-permissive internal environment through indirect gut–brain axis–related mechanisms, rather than directly promoting synaptic neurotransmission within the central nervous system.

In addition to neurochemical modulation, probiotics induced distinct changes in gut microbial composition. Phylum-level analysis showed that *Firmicutes* and *Bacteroidota* dominated the gut microbiota in all groups, with probiotic treatment slightly increasing the relative abundance of *Bacteroidota* and reducing Firmicutes. At the genus level, *Lactobacillus* and *Akkermansia* were markedly enriched in probiotic-treated mice, along with an upward trend in *Bacteroides fragilis* and *Bacteroides uniformis*. These taxa have been reported to produce neuroactive metabolites such as GABA and short-chain fatty acids (SCFAs), which can influence neuronal excitability and neurotransmitter synthesis indirectly through the gut-brain axis^26,27^. The selective enrichment of these beneficial taxa, while maintaining stable α-diversity indices, suggests that probiotics foster a microbiota configuration conducive to neurochemical balance without disrupting overall microbial homeostasis.

Several limitations of this study should be considered when interpreting the findings. First, sleep measurements were performed within a predefined recording window rather than across a continuous 24-hour period. To minimize variability and ensure comparability among experimental groups, sleep recordings were standardized to a fixed 4-hour recording duration under identical experimental conditions. While this design enabled controlled evaluation of sleep latency and sleep architecture, it does not fully capture circadian fluctuations or longer-term sleep–wake patterns. Accordingly, the present results primarily reflect sleep-related responses within the defined recording interval. In addition, although both low-dose (1 × 10⁹ CFU/day) and high-dose (1 × 10¹⁰ CFU/day) LM1063 exerted beneficial effects, the magnitude of improvement did not increase proportionally with dose escalation. This suggests that the in *vivo* sleep-promoting effects of LM1063 may not follow a strictly linear dose–response relationship, likely reflecting biological constraints and saturation effects in host–microbiota interactions. Second, while this study demonstrated that LM1063 administration modulates the expression of sleep-related receptor genes and associated neurochemical markers at the molecular level, it did not include an in-depth interrogation of the specific intracellular signaling pathways downstream of these receptors. As such, the current findings primarily delineate upstream molecular entry points through which LM1063 may influence sleep-related phenotypes and transcriptional responses, rather than providing a complete mechanistic cascade linking receptor activation to downstream gene regulatory networks and neural circuit modulation. Future studies integrating multi-omics profiling with targeted pharmacological or genetic perturbation strategies, together with electrophysiological and imaging-based approaches, will be essential to elucidate the precise intracellular signaling pathways and neural mechanisms that mediate probiotic-induced sleep modulation. Third, this study was conducted exclusively in male mice; therefore, it does not account for potential sex-dependent differences in sleep regulation or probiotic responsiveness. Given that sex hormones and sex-specific physiological factors can influence sleep–wake patterns, neurochemical signaling, and gut–brain axis interactions, the generalizability of the present findings to females remains to be determined. Future studies including both male and female animals will be important to assess potential sex-specific effects and to establish a more comprehensive understanding of probiotic-mediated modulation of sleep across sexes.

**Fig. 9:**
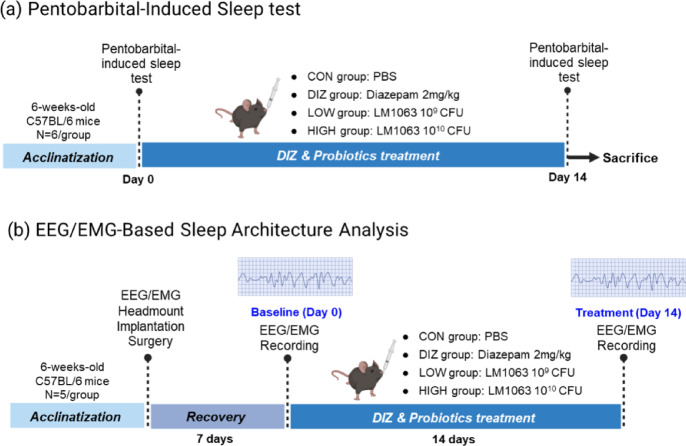
Experimental design and proposed mechanism of the sleep-promoting effects of *Limosilactobacillus reuteri* LM1063. Mice received vehicle (CON), diazepam (DIZ), or LM1063 at low or high doses, followed by pentobarbital-induced sleep testing and EEG/EMG recordings. LM1063 supplementation reduced sleep latency, increased total sleep time, and improved sleep architecture, with reduced wakefulness and a modest increase in REM sleep while maintaining stable NREM sleep. These effects were accompanied by coordinated modulation of GABAergic and serotonergic pathways and alterations in gut microbiota associated with neuroactive metabolite production, supporting a gut-brain axis-mediated contribution to optimized sleep patterns.

In conclusion, these findings highlight a dual mechanism by which probiotics promote sleep: (1) by directly modulating neurotransmitter-related gene expression and metabolite production, thereby strengthening GABAergic and serotonergic signaling, and (2) by indirectly enhancing neuroactive metabolite availability through the enrichment of GABA- and SCFA-producing gut bacteria. This integrative regulation through the gut-brain axis underscores the potential of probiotics as functional agents that promote restorative sleep and maintain neuro-intestinal homeostasis (Fig. 9).

## Materials and methods

### Preparation of probiotics

*Limosilactobacillus reuteri* LM1063 (LM1063) was provided in powder form by LactoMason Co., Ltd. (Seoul, Korea). Prior to use, the strain was cultured on de Man Rogosa Sharpe (MRS) agar containing 0.5% L-cysteine, and viable counts and species identity were confirmed by colony enumeration and re-identification. For animal experiments, the strain was stored at − 80 °C until administration. On each experimental day, LM1063 was freshly suspended in phosphate-buffered saline (PBS) to final concentrations of 1 × 10⁹ colony forming units (CFU) per 100 µL and 1 × 10¹⁰ CFU per 100 µL.

### Animals and experimental design

Male C57BL/6 mice (6 weeks old), obtained from Koatech (Pyeongtaek, Korea), were randomly assigned to experimental groups. For the pentobarbital-induced sleep test, six mice were used per group (*n* = 6/group), and for the EEG/EMG-based sleep architecture analysis, a separate cohort of five mice per group was used (*n* = 5/group). All procedures were approved by the Institutional Animal Care and Use Committee of Seoul National University (IACUC No. SNU-240501-5-1). All animal experiments were carried out in accordance with relevant institutional and national guidelines and regulations for the care and use of laboratory animals and are reported in compliance with the ARRIVE guidelines^28^. Mice were acclimated for one week under standardized laboratory conditions (temperature 23 ± 3 °C, relative humidity 50 ± 20%, 12 h light–dark cycle) with free access to chow and water.

**Fig. 10:**
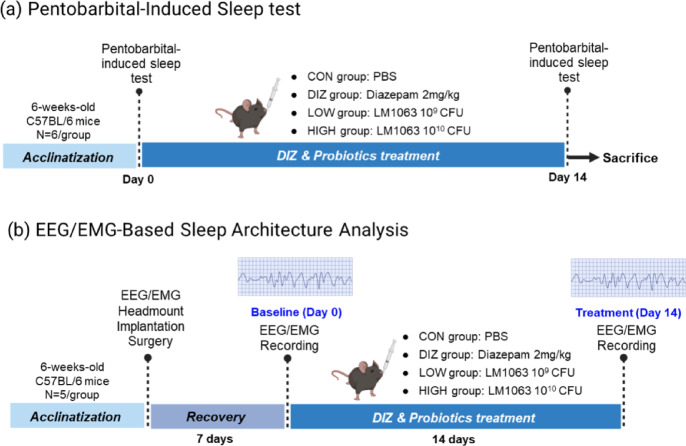
Schematic overview of animal experimental design. **(a)** Pentobarbital-Induced Sleep Test.** (b)** EEG/EMG-Based Sleep Architecture Analysis.

Following acclimation, animals were divided into four groups and administered daily treatments by oral gavage for two weeks: control (CON; PBS), positive control (DIZ; diazepam 2 mg/kg), low-dose LM1063 (LOW; 1 × 10⁹ CFU), and high-dose LM1063 (HIGH; 1 × 10¹⁰ CFU). Two independent experimental cohorts were established: one designated for the pentobarbital-induced sleep test and the other for sleep architecture analysis (Fig. 10).

### Pentobarbital-induced sleep test

The pentobarbital-induced sleep assay was conducted both at baseline (prior to treatment initiation) and on day 14 of the intervention period. Mice were fasted for 24 h before each test, with water provided *ad libitum*. On the test day, animals received the assigned treatment by oral gavage and were allowed to rest for 45 min, followed by intraperitoneal injection of pentobarbital sodium (45 mg/kg). After administration, mice were placed in individual cages, and sleep parameters were assessed. Sleep latency was defined as the time from pentobarbital injection to the loss of the righting reflex, and sleep duration was defined as the period from the loss of the righting reflex until its recovery. Throughout the procedure, animals were closely monitored to ensure their well-being, and any abnormal responses were promptly managed.

Following the sleep test, mice were rendered unconscious by carbon dioxide (CO₂) exposure, and blood was collected. Animals were then euthanized by continued CO₂ inhalation, and death was confirmed prior to tissue collection. Subsequently, tissues (intestine, brain) and feces were collected for downstream analyses. Fecal samples used for microbiota analysis were collected as freshly and spontaneously excreted pellets obtained directly from each individual mouse during a defined collection period before anesthesia, and intestinal contents collected after sacrifice were not used. Intestinal length was quantified from images taken immediately after dissection using ImageJ software (National Institutes of Health, USA).

### EEG/EMG-based sleep architecture analysis

EEG and electromyogram (EMG) headmount implantation were carried out following the manufacturer’s protocol (Pinnacle Technology, USA) and established methodological references^29–31^. Mice were anesthetized via intraperitoneal injection of a ketamine–xylazine cocktail prepared by mixing ketamine, xylazine, and distilled PBS at a ratio of 7:1:17 (100–120 µL per mouse) and were then positioned in a stereotaxic apparatus (David Kopf Instruments, USA). After scalp incision and removal of the periosteum, stainless steel screw electrodes were fixed to the skull to record cortical EEG activity, and insulated EMG wires were inserted into the neck muscles to monitor muscle tone. The headmount assembly was secured to the skull with dental cement, and the incision was sutured. Following a one-week postoperative recovery, mice were habituated to the recording chamber to minimize stress before data collection. EEG and EMG recordings were obtained at baseline (before treatment initiation) and on day 14 of the experimental period. Sleep-wake stages, including wakefulness, non-rapid eye NREM sleep, and REM sleep, were classified in 10-second epochs using Sirenia^®^ Sleep Pro Software (Pinnacle Technology, USA) according to standard scoring criteria. Quantitative parameters of sleep architecture, such as total sleep time and the relative proportion of each stage, were analyzed for group comparisons. At the completion of EEG/EMG recordings, mice were euthanized by CO₂ inhalation using a dedicated chamber, followed by confirmation of death.

### RNA extraction and quantitative reverse transcription PCR (RT-qPCR)

Brain and intestinal tissues were homogenized in TRIzol reagent (Invitrogen, USA) using a bead homogenizer, and total ribonucleic acid (RNA) was purified with the RNeasy Mini Kit (Qiagen, Germany). RNA concentration and purity were assessed spectrophotometrically, and 1 µL of RNA was reverse-transcribed into complementary DNA (cDNA) using the iScript cDNA Synthesis Kit (Bio-Rad Laboratories, USA). Subsequently, qRT-PCR was performed with gene-specific primers using iQ™ SYBR Green Supermix on a CFX96 Real-Time PCR System (Bio-Rad, USA), with GAPDH as the reference gene. Fold changes were determined using the 2^–ΔΔCT^ method, and primer sequences are listed in Supplementary Table 1.

### Serum enzyme-linked immunosorbent assay (ELISA)

Immediately after sacrifice, blood samples were collected and centrifuged at 1,500 rpm for 15 min to obtain serum, which was aliquoted and stored at −80 °C until analysis. Serum concentrations of glutamate (MyBioSource, USA; Cat. No. MBS2601720), γ-aminobutyric acid (GABA; MyBioSource, USA; Cat. No. MBS260709), and serotonin (MyBioSource, USA; Cat. No. MBS1601042) were quantified using commercial ELISA kits according to the manufacturers’ protocols. Standards and samples were assayed, and absorbance was measured at 450 nm using a microplate reader. Concentrations were calculated from standard curves generated for each analyte.

### Fecal DNA Extraction and 16S rRNA Gene sequencing

To characterize changes in the gut microbiota of mice, 16S rRNA gene-based metagenomic sequencing was performed using fecal samples. Fecal samples were collected weekly from freshly and spontaneously excreted fecal pellets obtained directly from each mouse during a defined collection period and were immediately snap-frozen and stored at − 80 °C until analysis. Genomic deoxyribonucleic acid (DNA) was extracted using the DNeasy PowerSoil Pro Kit (Qiagen, Germany) according to the manufacturer’s protocol. The concentration and integrity of extracted DNA were assessed using a spectrophotometer (Molecular Devices, USA) and agarose gel electrophoresis. The primer set used for amplifying the V3-V4 region of the 16S rRNA gene was as follows: forward primer, 5′-TCG TCG GCA GCG TCA GAT GTG TAT AGA CAG GTG CCA GCM GCC GCG GTA A-3′; and reverse primer, 5′-GTC TCG TGG GCT CGG AGA TGT GTA TAA GAG ACA GGG ACT ACH VGG GTW TCT AAT-3′. Amplicon sequencing was conducted by Sanigen Inc. (Anyang, Korea) on the Illumina NextSeq platform (Illumina, USA) with 2 × 300 bp paired-end reads. Raw sequence data were demultiplexed, quality-filtered, and denoised to remove adapters, low-quality bases, and chimeric sequences. Taxonomic classification was performed after excluding mitochondrial and chloroplast sequences, and microbial community diversity was evaluated using α-diversity and β-diversity analyses. Differentially abundant taxa among groups were identified using linear discriminant analysis effect size (LEfSe) with a threshold logarithmic LDA score of 2.0.

### Statistical analysis

In vitro experiments were performed in three independent replicates. For in vivo experiments, data were obtained from independent cohorts with sample sizes indicated in the figure legends. Specifically, EEG/EMG sleep scoring was performed independently by two investigators to ensure reliability. Results are expressed as the mean ± standard error of the mean (SEM). Statistical significance was assessed by one-way analysis of variance (ANOVA) followed by Tukey’s post hoc test using GraphPad Prism version 10.0 (GraphPad Software, USA). Significance levels were defined as *p < 0.05, **p < 0.01, and ***p < 0.001. For analysis involving baseline and post-treatment measurements, two-way ANOVA with Group and Time as factors was additionally applied to evaluate main effects and interaction effects, as appropriate.

## Supplementary Information

Below is the link to the electronic supplementary material.

Supplementary Material 1

## Data Availability

The raw metagenomic sequencing datasets supporting this study are available in the NCBI Sequence Read Archive (SRA) under BioProject accession number PRJNA1322386. Access to the data will be provided upon request.
