# Supplementary material for: Hypnotic and sleep-promoting effects of Limosilactobacillus reuteri LM1063 on pentobarbital-induced sleep and electroencephalogram analysis in mice
Source: Sci Rep. 2026 Mar 9;16:12820. doi: 10.1038/s41598-026-42833-0 (PMC13096653; doi:10.1038/s41598-026-42833-0)

**Supplementary materials**

**Hypnotic and Sleep‑Promoting Effects of *Limosilactobacillus reuteri* LM1063 on Pentobarbital-induced Sleep and Electroencephalogram Analysis in Mice**

Min Gyeong Kim^a,†^, Eunsol Seo^a,†^, Ju Young Eor^b,†^, Anna Kang^a^, Tae Rahk Kim^b^, Minn Sohn^b^, and Younghoon Kim^a,*^

^a^Department of Agricultural Biotechnology and Research Institute of Agriculture and Life Science, Seoul National University, Seoul 08826, Korea

^b^LactoMason Co., Ltd., Jinju 52840, Korea

^†^These authors contributed equally to the manuscript

*To whom correspondence should be addressed: ykeys2584@snu.ac.kr

**Supplementary Table S1.** The list of primer sequences used for qPCR

| **Gene name** | **Forward primer (5` - Seq - 3`)** | **Reverse primer (5` - Seq - 3`)** | **Reference** |
| --- | --- | --- | --- |
| *GAPDH* | AATGGTGAAGGTCGGTGTGA | ACTGTGCCGTTGAATTTGCC | In this study |
| *GABAARα2* | TTACAGTCCAAGCCGAATGTCCC | ACTTCTGAGGTTGTGTAAGCGTAGC | (Baek et al., 2024) |
| *GABAAR* | AAAAGTCGGGGTCTCTCTGAC | CAGTCGGTCCAAAATTCTTGTGA | In this study |
| *BDNF* | TACCTGGATGCCGCAAACAT | TGCTTCAGTTGGCCTTTGGA | In this study |
| *5-HT1AR* | CCGTGAGAGGAAGACAGTCTAAGA | GGTTGAGCAGGGAGTTGGAGTAG | (Baek et al., 2024) |
| *5-HT2AR* | ACATCCTCGGTCACCTCCATTA | CCCTAGCGGCCATGAGTTTC | (Baek et al., 2024) |
| *5-HT3AR* | *GTGATAAGCCTCGCTGAGACC* | CGCATCTCATCCCGCTTCT | (Taverniti et al., 2021) |
| *5-HT4R* | *GATGCCCTTTGGTGCCAT* | CAGCAGATGGCGTAATACCTG | (Taverniti et al., 2021) |
| *5-HT7R* | GGCTACACGATCTACTCCACCG | CGCACACTCTTCCACCTCCTTC | (Baek et al., 2024) |
| *SERT* | CAAAACCAAGAACCAAGAG | CATAGCCAATGACAGACAG | (Choi et al., 2025) |

**Reference**

Baek, J.-S., Lee, D.-Y., Han, S.-W. , Kim, D.-H., 2024. A probiotic NVP1704 alleviates stress-induced sleeplessness/depression-like symptoms in mice by upregulating serotonergic and GABAergic systems and downregulating NF-κB activation. Letters in Applied Microbiology 77, ovae065. <https://doi.org/10.1093/lambio/ovae065>.

Choi, H., Kwak, M.-J., Choi, Y., Kang, A. N., Mun, D., Eor, J. Y., Park, M. R., Oh, S. , Kim, Y., 2025. Extracellular vesicles of Limosilactobacillus fermentum SLAM216 ameliorate skin symptoms of atopic dermatitis by regulating gut microbiome on serotonin metabolism. Gut Microbes 17, 2474256. <https://doi.org/10.1080/19490976.2025.2474256>

Taverniti, V., Cesari, V., Gargari, G., Rossi, U., Biddau, C., Lecchi, C., Fiore, W., Arioli, S., Toschi, I. , Guglielmetti, S., 2021. Probiotics modulate mouse gut microbiota and influence intestinal immune and serotonergic gene expression in a site-specific fashion. Frontiers in Microbiology 12, 706135. <https://doi.org/10.3389/fmicb.2021.706135>

**Supplementary Fig. S1.** Relative mRNA expression levels of intestinal barrier markers (*Muc2*, *Occludin*, *Claudin-1*, and *Zo-1*) in colon tissue following probiotic administration.


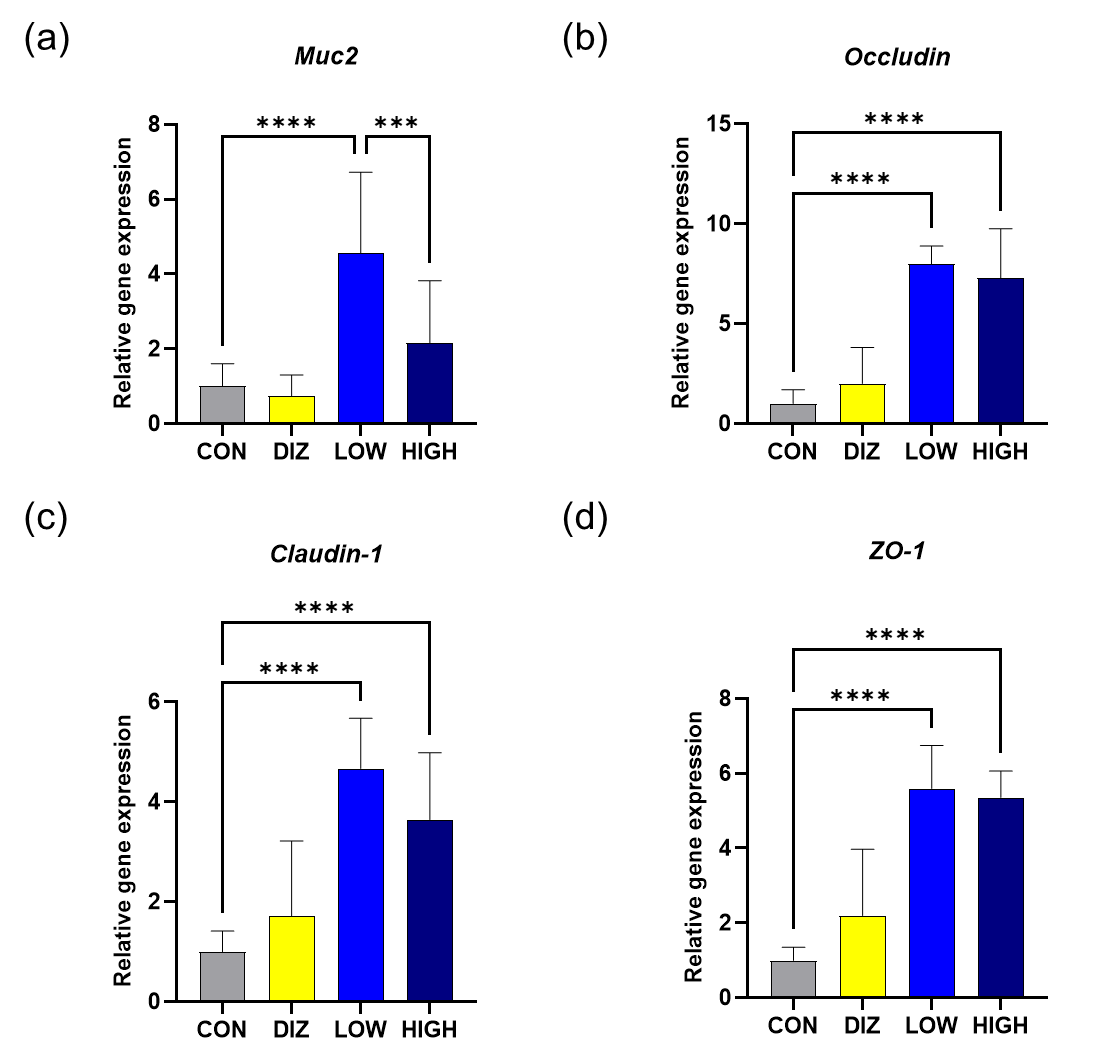

Supplement: Supplementary file 1 — Supplementary Material 1 [file 41598_2026_42833_MOESM1_ESM.docx]
